# Arrhythmia-Induced Cardiomyopathy: Predictors of Improvement in Left Ventricular Systolic Function After Catheter Ablation

**DOI:** 10.3390/jcm14051636

**Published:** 2025-02-28

**Authors:** Marisa Varrenti, Eleonora Bonvicini, Matteo Baroni, Lorenzo Gigli, Marco Carbonaro, Ilaria Garofani, Giulia Colombo, Sara Vargiu, Valentina De Filippo, Federica Giordano, Raffaele Falco, Antonio Frontera, Roberto Menè, Alberto Preda, Patrizio Mazzone, Fabrizio Guarracini

**Affiliations:** 1 Electrophysiology Unit, De Gasperis Cardio Center, Niguarda Hospital, 20162 Milan, Italy; matteo.baroni@ospedaleniguarda.it (M.B.); lorenzo.gigli@ospedaleniguarda.it (L.G.); marco.carbonaro@ospedaleniguarda.it (M.C.); ilaria.garofani@ospedaleniguarda.it (I.G.); giulia.colombo@ospedaleniguarda.it (G.C.); sara.vargiu@ospedaleniguarda.it (S.V.); valentina.defilippo@opsedaleniguarda.it (V.D.F.); federica.giordano@ospedaleniguarda.it (F.G.); raffaele.falco@ospedaleniguarda.it (R.F.); antonio.frontera@ospedaleniguarda.it (A.F.); roberto.mene@ospedaleniguarda.it (R.M.); alberto.preda@ospedaleniguarda.it (A.P.); patrizio.mazzone@ospedaleniguarda.it (P.M.); fabrizio.guarracini@ospedaleniguarda.it (F.G.); 2 Department of Cardiology, Santa Chiara Hospital, 38122 Trento, Italy

**Keywords:** arrhythmia-induced cardiomyopathy, cardiac magnetic resonance, catheter ablation, heart failure, left ventricular systolic function

## Abstract

**Background:** Arrhythmia-induced cardiomyopathy (AIC) is a relatively common yet poorly understood cardiac condition that occurs when arrhythmias cause left ventricular systolic dysfunction, which can be reversed with the resolution of the arrhythmia. Catheter ablation serves as a cornerstone treatment for eliminating the arrhythmic trigger. However, the variability in left ventricular recovery following ablation highlights the need to identify reliable predictors of reverse remodeling. **Methods**: This review explores key studies on AIC patients undergoing catheter ablation, primarily derived from observational cohorts, to identify significant predictors of left ventricular function recovery. **Results**: While echocardiography and cardiac magnetic resonance imaging remain the primary diagnostic modalities, additional insights have emerged from electrocardiograms and laboratory biomarkers. Despite these advancements, a comprehensive framework for identifying optimal candidates for ablation remains lacking. **Conclusions**: By synthesizing existing evidence, this review aims to pinpoint the most robust predictors of systolic recovery in AIC patients following ablation.

## 1. Introduction

Cardiac arrhythmias and heart failure are significant cardiological conditions that often coexist and exacerbate each other. Persistent arrhythmias leading to impaired left ventricular function are classified as arrhythmia-induced cardiomyopathy (AIC). Both atrial and ventricular arrhythmias can contribute to AIC, with atrial fibrillation being the most frequent cause [1,2]. However, data on the incidence and prevalence of AIC remain inconsistent, and limited information is available regarding its long-term prognosis. In patients with AIC, ventricular dysfunction arises from a complex pathophysiology. Impaired ventricular rate reduces blood flow and causes ventricular dyssynchrony, which leads to myocyte remodeling, electrical disturbances, and neurohormonal activation. This cascade ultimately results in a loss of contractile function [3]. Despite understanding these mechanisms, many questions remain unanswered. For instance, why do some patients develop ventricular dysfunction under similar heart rates or arrhythmic burdens while others remain unaffected? Furthermore, not all patients with AIC achieve full recovery of left ventricular function after arrhythmia resolution. Patient-specific factors, including a potential genetic predisposition, appear to influence reverse remodeling and clinical outcomes [4]. Identifying patients with a higher likelihood of ventricular function recovery is crucial for optimizing disease management and tailoring therapeutic strategies such as catheter ablation. While predictors of left ventricular recovery have been explored in several observational studies across different domains, a more systematic approach is needed to consolidate the evidence and guide clinical practice.

## 2. The Aims and Methods of the Review

The aim of our study was to conduct a comprehensive literature review to identify predictors of left ventricular function recovery following arrhythmia ablation therapy in patients diagnosed with AIC. 

We conducted our literature search using major scientific platforms, including PubMed, Embase, and the Cochrane Central Register of Controlled Trials, with the following combined keywords: tachycardia, cardiomyopathy, ventricular dysfunction, heart failure, ablation. The results were independently analyzed by two reviewers, with an initial selection based on titles and abstracts. A manual search through references and citations was also performed to identify additional relevant articles. The full texts of the selected studies were independently reviewed by each reviewer for final inclusion, with disagreements resolved through discussion. The reviewers examined the study populations, baseline patient characteristics, post-ablation outcomes, and predictors of recovery separately. The primary objective was to identify the strongest predictors of left ventricular ejection fraction (LVEF) recovery in patients with AIC following ablation therapy. To streamline the analysis, we divided our research and results into two major categories: supraventricular arrhythmias and ventricular arrhythmias. 

## 3. Supraventricular Arrhythmias

Supraventricular arrhythmias (SVTs) are the primary contributors to AIC [5]. The irregular heart rate (HR) and the loss of atrial kick, which increase filling pressures and lead to diastolic dysfunction, are believed to underlie the systolic dysfunction observed in AIC [6]. In adults, the most common causes of AIC are atrial fibrillation (AF) and atrial flutter (AFL) [7], while in children, atrial tachycardia (AT) and permanent junctional reciprocating tachycardia are predominant triggers [8]. Less frequently, AV nodal re-entrant tachycardia and sustained sinus tachycardia are identified as causes. The incidence and prevalence of AIC remain challenging to estimate due to diagnostic uncertainties. However, studies suggest an incidence of 8–10% in patients with AT [9,10], 5–8% in those with AF [2], and 5–14% in patients with AFL [2,11]. Therapeutic strategies for AIC focus on normalizing HR and restoring sinus rhythm, achievable through antiarrhythmic drugs, electrical cardioversion, or catheter ablation (CA) [12]. For SVTs, recent evidence highlights that rhythm control strategies outperform rate control in reducing mortality and hospitalizations [13,14,15]. Among rhythm control options, CA has demonstrated superior outcomes by maintaining sinus rhythm without the side effects associated with antiarrhythmic drugs [16,17,18]. In patients with AIC, CA was shown to improve LVEF and enhance quality of life while presenting a low risk of adverse events [19,20]. Consequently, the European Society of Cardiology (ESC) guidelines recommend CA as a Class I treatment (Level of evidence B) for SVTs responsible for AIC [21,22].

### Predictors of Left Ventricular Systolic Function Improvement

Due to the difficulty in the diagnosis of AIC, selection of the patient who will benefit the most from a procedure of CA is challenging. Identifying predictors of EF recovery could help to better resource targeting in order to offer CA to the most suitable patients, leaving other solutions to the rest. Only a limited number of studies, which were mainly observational ones, have been conducted in this direction, but robust data have been highlighted, providing even some predictive models.

Pre-procedural left ventricular (LV) end diastolic volume and diameter have proved to be the most recognized predictors of left ventricular systolic function improvement. In two recent studies, a total of ninety-six and a hundred patients, respectively [23,24], with AF and LVEF < 50% underwent CA, and reduced LV end diastolic diameter (LVEDD) was pointed out as an independent predictor of LVEF improvement (*p* = 0.0002 and *p* = 0.049). More specifically, different analyses recognize LVEDD as inferior to 53 mm [25,26] or to 59 mm [27] in patients with a better response to CA, while a reduced LV end diastolic volume (LVEDV) and end systolic volume (LVESV) was found in patients after CA with reverse remodeling compared with those without (112 ± 44 vs. 151 ± 43 and 69 ± 33 vs. 102 ± 52; *p* = 0.0273 and 0.0443) [28]. Similar results were identified in other studies involving not only AF patients [29,30,31,32], but even AFL [33] and other SVTs [34,35]. Moreover, in a cohort of sixty patients with AFL, a pre-ablation LVEDV ≥ 137 mL was associated with no improvement of LV performance after ablation [36], while Moore et al. demonstrated that baseline LVEDD (HR 0.86, *p* = 0.008) was predictive for LVEF normalization (*p* = 0.005) in eighteen children with SVTs and a median age of 4 years old [37]. Overlapping results were obtained with methods other than echocardiography, like cardiac magnetic resonance (CMR) [38]. Along the same lines, a lower pre-procedural EF was proved to correlate to a worse response to CA [33,39]. In particular, different studies seem to agree on the fact that patients with HFrEF (LVEF < 40%) exhibited lower proportions of LV systolic function recovery than patients with HFmrEF (LVEF 40–50%) [31,34,40]. These results suggest that AIC takes place before a significant LV remodeling and a greater LVEF reversibility must be correlated with a less impaired ventricle. Therefore, the longer the tachyarrhythmia lasts, the less likely the recovery of LV function becomes. Of note, the only data from speckle analysis that were identified in AIC evaluation was the relative apical longitudinal strain ratio (RALSR) that, with a value of 0.61, was a specific and sensitive (71–90%) predictor of LV systolic recovery [35].

Similar considerations can be made regarding the left atrium (LA). Left atrial dilation tends to increase with arrhythmia recurrence, reflecting the severity of the disease. A smaller left atrium was suggested as a potential predictor of LVEF recovery. For instance, Bergonti et al. [41] reported that an indexed left atrial volume (LAVI) below 50 mL/m^2^ (OR 9.1) correlates with a favorable response to CA. In addition to atrial volume and systolic function, the assessment of diastolic function is also important, as it was shown to correlate with reverse remodeling following cardiac resynchronization therapy [42] and similarly after CA [35]. Specifically, studies have identified optimal thresholds for septal e’ velocity lower than the standard 7 cm/s, with cutoffs of 5.4 cm/s and 6.3 cm/s proposed in two separate studies [25,32].

The recent guidelines recommend CMR in all patients with cardiomyopathy at initial evaluation (Class of recommendation I, Level of evidence B) [43]. While no indication has been drawn for AIC induced by supraventricular arrhythmias, it was demonstrated that the study of myocardial fibrosis, as assessed by late gadolinium enhancement (LGE), plays a dual role in the context of AIC. It can help exclude other causes of left ventricular dysfunction and identify myocardial damage associated with advanced disease. A history of ischemic heart disease (IHD) was shown to negatively predict LV recovery [33,44,45], as reverse remodeling is challenging to achieve despite optimal medical therapy and arrhythmia reduction. LGE patterns, such as subendocardial or transmural enhancement, are negative predictors of LV recovery. Similarly, other LGE patterns characteristic of specific cardiomyopathies may also indicate poor outcomes. In an observational study involving 43 patients, Vera et al. [39] demonstrated that patients with AIC who responded to CA with LV remodeling were less likely to exhibit LGE (16% vs. 61%, *p* = 0.004). In these cases, LGE was typically localized in the mid-myocardium with a non-specific distribution. Other studies have corroborated the absence of LGE as a predictor of LVEF recovery [38,46,47]. Notably, the CAMERA study [48]—the only randomized clinical trial on this topic—confirmed that the presence of LGE was associated with a lack of LVEF normalization at six months (*p* = 0.0296). Additionally, in this cohort, patients with positive LGE had higher LVEDV, linking advanced disease to poor outcomes. Quantification of extracellular volume using T1 mapping sequences has also shown promise in predicting reverse remodeling and could serve as an alternative when CMR is contraindicated [28]. Furthermore, assessing myocardial fibrosis in the left atrium may help identify responders to CA. Kirstein et al. [49], in a study involving 103 patients with reduced LVEF (median: 33%; range: 25–38%), demonstrated that fibrosis covering more than 35–40% of the left atrial surface was associated with a lack of LVEF improvement following CA for AF. Similarly, Zhao et al. [27] found that the absence of low-voltage zones in the left atrium independently predicted LVEF improvement after AF ablation. 

Elevated troponin levels are indicator of damage to the myocardial cells and it was proved that in heart failure (HF), patients with arrhythmias are associated with worse outcomes [50]. This notion was at the base of the studies conducted by Aoyama et al. [23,26,51] that established that healthier myocardium with less fibrosis depicted by low pre-procedural troponin level responds very well to CA. In detail, a threshold of 12 pg/mL for high sensibility troponin T (hs-TnT) and of 11 pg/mL for troponin I (TnI) was identified as predictors of LV recovery (sensitivity/specifity 90.0%/76.7%; 79.1%/76.5%; positive predictive value/negative predictive value: 89.5%/59.1%; 56.3%/95.8%, respectively). Among the cardiac biomarkers, NT-proBNP also seems to have a role as a predictor of LVEF recovery [28].

Valuable information could also be retrieved through another more practical method, which is electrocardiogram (ECG). First of all, the ventricular rate of the tachyarrhythmia has a weight in deciding the fate of LV recovery: it was suggested that a ventricular response of 80–100 beats/min or greater defines a better improvement in LV function [11,31,33]. These data are very important because they highlight how ventricular response is a very important factor in establishing systolic dysfunction. Likewise, persistent AF, which has a more stressful impact on the ventricle, leads to a more important LV dysfunction and is associated with better LV recovery [30,41,46]. Intraventricular conduction delays that express themselves on the surface ECG with a wide QRS are known to be correlated in cardiomyopathies to myocardial injury and fibrosis [52,53]. In this perspective, it was demonstrated that wider QRS could be negative predictors of LV recovery in AIC [24,39,41]. Vera et al. proposed a cutoff for QRS duration ≥ 100 ms (*p* = 0.027) [39], while in the ANTWOORD study, Bergonti et al. suggested a QRS ≥120 ms (*p* < 0.001) [41]. Similarly, atrial fibrosis that impairs atrial conduction has been well portrayed by particular data extrapolated from the surface ECG, the maximum P-wave duration (max PWD), and the P-wave terminal force in lead V1 (PTFV1) [54]. Along these lines, Doi et al. [55] proved that PTFV1 was a reliable predictor of complete response to CA.

Lastly, on top of the laboratory and instrumental evaluation, a clinical assessment of the patient is undetachable. In fact, younger age [45,51], better functional status [40], and lower CHA2DS2-VASc [51] were also identified as independent predictors of LV recovery in AIC.

Genetic predisposition cannot be ruled out, but up until now, only a polymorphism of the HCN4 gene has been identified as a possible marker for AIC in patients with AF [56].

Table 1 summarizes the aforementioned studies about the argument.

## 4. Ventricular Arrhythmias

Ventricular premature complexes (PVCs) are the most common benign ventricular arrhythmias, affecting over 75% of individuals with healthy hearts [57]. Although PVCs generally follow a benign course, they can lead to left ventricular dysfunction and, consequently, the development of AIC. Animal studies have explored the molecular mechanisms underlying left ventricular dysfunction in AIC patients. Key mechanisms include an imbalance between increased metabolic demands and insufficient blood flow due to hemodynamic impairment, primarily caused by ventricular dyssynchrony and secondarily by elevated HR. Genetics may also contribute to AIC development in patients with ventricular arrhythmias; however, this area remains underexplored [1].

### 4.1. PVC Burden and Its Impact on AIC

The PVC burden over 24 h appears to be the most significant predisposing factor for AIC. Although no universally validated cutoff exists, Hasdemir et al. [58] analyzed data from 249 patients with a history of ventricular arrhythmias (PVCs and/or sustained or non-sustained ventricular tachycardias). Among these, 17 patients (6.8%) developed AIC, with a PVC burden ≥ 16% emerging as a strong determinant of left ventricular dysfunction (LVEF ≤ 50%), showing 100% sensitivity and 87% specificity. Following CA, the mean LVEF improved significantly from 38 ± 7% to 53 ± 7%. Similar findings were reported in other studies for PVC burdens > 20% [59,60].

### 4.2. PVC Origin and Ventricular Dysfunction

The origin of PVCs also influences their impact on ventricular function. PVCs originating from the right ventricular outflow tract (RVOT) appear to cause more severe ventricular dysfunction compared to those from the LV. This may be due to greater dyssynchrony caused by RVOT stimulation resembling a bundle branch block. In a study by Thibault Johan Mørk et al. [61], 131 patients with idiopathic ventricular arrhythmias underwent CA. Among them, 16 patients with left ventricular dysfunction experienced complete recovery after ablation (LVEF improved from 44% [IQR: 31–47] to 60% [IQR: 60–60]). Interestingly, arrhythmias originating from the LV were more commonly associated with AIC, indicating that LV-originating PVCs pose a higher risk for developing cardiomyopathy.

### 4.3. Other Predictors of AIC Development

Additional predictors of AIC include QRS duration > 140 ms and male gender, both identified as independent risk factors for developing AIC [62]. The improper identification of PVC prevalence in the general population, variability in predisposing factors, and retrospective diagnoses have likely led to the underestimation of PVC-related cardiomyopathy prevalence.

### 4.4. Ablative Therapy for PVC-Related AIC

As with SVTs, CA is a valuable treatment option for reducing or eliminating PVC burden and improving ventricular function. Yarlagadda et al. [63] studied 27 patients with repetitive monomorphic PVCs originating from the RVOT. Among these, eight patients (30%) had left ventricular dysfunction (LVEF < 45%), and seven underwent successful ablation. This resulted in the complete recovery of LVEF, improving from 39 ± 6% to 62 ± 6% (*p* = 0.017).

### 4.5. Predictors of Left Ventricular Systolic Function Improvement

In the context of CA, predictors of LVEF recovery were studied to develop a more personalized approach for treating PVC-related cardiomyopathy. Table 2 summarizes the major studies on the argument. Gopinathannair et al. [64] analyzed 243 patients with AF, AT, and PVCs, focusing on predictors of LVEF recovery. Their findings showed that left ventricular systolic function improved regardless of arrhythmia type or duration. However, patients with PVCs had lower baseline LVEF and less recovery following ablation. Similarly, earlier studies identified lower baseline LVEF as a negative predictor of improvement [65]. Wojdyła-Hordyńska et al. [66] reported data on 109 patients referred for ablation due to a history of PVCs with an extrasystolic burden > 10,000 in 24 h. Ablation effectively reduced the PVC burden in 85.4% of cases after a single procedure. In patients with ventricular dysfunction, mean LVEF improved from 37% ± 8% to 48 ± 13%. Recovery was more significant in patients with dilated cardiomyopathy (LVEF increased from 39.73% to 47.72%, *p* < 0.001) or idiopathic cardiomyopathy (LVEF increased from 57.9% to 63.49%, *p* < 0.001). In contrast, no significant changes were observed in patients with IHD. Multivariate analysis identified an extrasystolic burden > 20,000/24 h as the sole predictor of EF improvement. Similarly, a prospective multicenter study involving 80 patients with PVCs found that ablation was successful in 53 patients (66%). These individuals experienced an improvement in LVEF from 33.7 ± 8% to 45.8 ± 10.9% at 12 months post-treatment. Regardless of underlying structural heart disease, improvements in HF parameters correlated strongly with baseline PVC burden: a PVC burden ≥ 13% had a sensitivity of 100% and specificity of 85% for predicting an EF recovery of at least 5% from baseline.

Not only the burden but also the origin of PVCs influences the likelihood of LVEF recovery. Dyssynchrony caused by RV-originating PVCs is particularly harmful and wasassociated with an increased risk of irreversible cardiomyopathy. Studies have shown higher incidences of EF recovery after ablation targeting LV substrates compared to RV ones [67]. Deyell et al. [68] studied data from patients with left ventricular dysfunction (LVEF ≤ 50%) and a PVC burden ≥ 10% over 24 h who underwent ablation. Among the cohort of 114 patients, 48 had LV dysfunction; ablation was performed on 37 patients. Of these, 24 experienced reversible LV dysfunction with an average EF improvement of 17.5% (IQR: 10–40%), 2 had partially reversible dysfunction (EF improvements of 10% and 25%), and 11 had irreversible LV dysfunction. Multivariate analysis revealed that increased QRS duration was significantly associated with non-reversible or partially reversible LV dysfunction; QRS duration > 170 ms was particularly predictive of poor recovery outcomes.

### 4.6. Imaging in Predicting Recovery of Left Ventricular Function

Echocardiography plays a critical role in identifying patients at risk of failing to recover ventricular systolic function after ablative treatment. Kusunose and colleagues [35] conducted a retrospective study on 71 patients with AIC, defined as left ventricular dysfunction (LVEF ≤ 50%) without other identifiable causes. Patients were divided into two groups based on arrhythmia type—supraventricular or ventricular—and were evaluated six months after initiating either medical therapy or ablative treatment. The primary endpoint was defined as an improvement in LVEF of at least 10%. A control group of 30 patients was also included for comparison. Of the 71 patients, 27 underwent electrophysiological studies and ablation, while 23 received medical therapy. LVEF improvement was observed in 40 patients. Those who did not experience significant improvement had larger baseline left ventricular size, reduced LVEF, elevated E/e ratio, and higher tricuspid regurgitant pressure gradient (TR-PG). Additionally, these patients exhibited altered strain values, including significantly lower baseline longitudinal strain and higher RALSR. On multivariate analysis, LVEF recovery was strongly correlated with TR-PG and RALSR. A RALSR cutoff value of 0.61 demonstrated high sensitivity (71%) and specificity (90%) for predicting functional recovery of left ventricular systole.

In PVC-induced cardiomyopathy, CMR is suggested as part of the diagnostic pathway by ESC guidelines (Class of recommendation IIa, Level of evidence B). It was demonstrated that CMR also provides valuable insights into predicting LVEF recovery. Hasdemir et al. [69] investigated the presence of LGE in a cohort of 27 patients with AIC caused by ventricular arrhythmias, including PVCs, sustained ventricular tachycardias, and non-sustained ventricular tachycardias. Within six weeks of ablation, significant improvement in LVEF was observed in 22 out of 27 patients (from 39.8 ± 8.9% to 54.8 ± 7.9%, *p* < 0.001). Among the 19 patients who underwent CMR with contrast enhancement, LGE was detected in only 1 patient who experienced EF recovery (5%) but in 4 out of 5 patients with persistent EF depression (80%). Although further studies are required to confirm the predictive value of LGE for non-recovery after ablation, the authors concluded that CMR is essential for identifying patients with underlying cardiomyopathy who may have a lower likelihood of functional recovery after ablation.

**Table 2:** Predictors of EF recovery in patients with AIC and ventricular arrhythmias.

Author, Year	N. Patients	Type of Arrhythmia	Basal EF vs. Follow-Up EF	Duration of Follow-Up	Baseline Predictor of LV Recovery
Yarlagadda et al., 2005 [63]	27	PVC from RVOT	39 ± 6% vs. 62 ± 6%;	8 ± 10 months	
Wojdyła-Hordyńska et al., 2017 [66]	109	PVC from LVOT and RVOT	37 ± 8% vs. 48 ± 13%	6 months	PVC Burden ≥ 20,000/24 h and no history of IHD
Mørk et al., 2014 [61]	131	PVC	44% (IQR: 31–47) vs. 60%	8 ± 10 months	PVC not originating from LVOT
Niwano et al., 2009 [70]	281	PVC from LVOT and RVOT	-	67.2 ± 20.4 years	Lower PVC prevalence and higher LVEF
Deyell et al., 2012 [68]	114	PVC from LV and RV	-	10.6 months	Narrower PVC QRS
Hasdemir et al., 2012 [64]	27	PVC VT	39.8 ± 8.9% vs. 54.8 ± 7.9%	6 months	Absence of LGE
Gopinathannair et al., 2021 [64]	243	PVC (31%)	25.1 ± 6.7 vs. 40.7 ± 10.9	5.2 ± 2.5 months	Higher LVEF
Kusunone et al., 2017 [35]	71	PVC and nsVT (10%)	39 ± 8% vs. 53 ± 12%	6 months	Lower LV size, E/e’, TR-PG, higher LVEF and LS RALSR ≤ 0.16
Panela et al., 2013 [71]	80	PVC	33.7 ± 8% vs. 45.8 ± 10.9%	12 months	Burden PVC ≥ 13%

LGE = late gadolinium enhancement; LS = longitudinal strain; LV = left ventricular; LVEF = left ventricular ejection fraction; LVOT = left ventricular outflow tract; nsVT = non sustained ventricular tachycardia; PVC = ventricular premature beats; RALSR = relative apical longitudinal strain ratio; RVOT = right ventricular outflow tract; TR-PG = tricuspid regurgitant pressure gradient; VT = ventricular tachycardia.

### 4.7. Pediatric Population

An international multicenter study [37] examined pediatric patients aged ≤18 years with incessant tachyarrhythmia and ventricular dysfunction (EF < 50%), excluding those with congenital heart disease or suspected primary cardiomyopathy. Among the 81 patients studied, AT and reciprocating junctional tachycardia were the most common arrhythmias, while ventricular tachycardia was identified as the trigger arrhythmia in 7% of cases. Management strategies included medical therapy alone in 26 patients (32%), medical therapy followed by ablation in 27 patients (33%), and ablation alone in 28 patients (35%). On multivariate analysis, predictors of LVEF recovery included younger age, lower HR at presentation, circulatory support during treatment, and baseline EF.

## 5. Predictive Model

**Figure 1:**
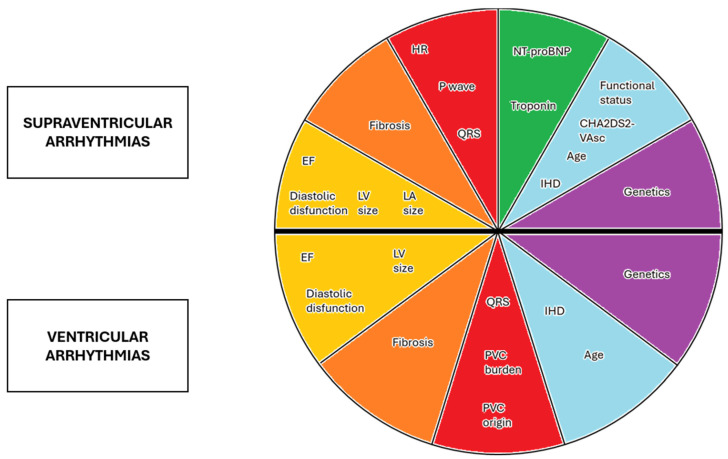
Predictors of EF recovery in AIC and supraventricular and ventricular arrhythmias. EF = ejection fraction; HR = heart rate; IHD = idiopathic heart disease; LA = left atrium; LV = left ventricle; PVC = premature ventricular contraction.

Several independent predictors of LV recovery have emerged in the aforementioned studies and are summarized in Figure 1, but what emerges is the necessity to develop a predictive model able to bring together different aspects of the patient to lead to a conscious choice. Some proposals have already been made for SVTs in the Bergonti et al. [41] and Vera et al. [39] studies, which have suggested two predictive models based on ECG and echocardiography or CMR data, respectively. A similar approach could also be proposed for PVC-induced cardiomiopathy, but further studies are needed to provide a validated model in both fields.

## 6. Conclusions

Patients with both ventricular and supraventricular arrhythmic events may experience left ventricular dysfunction, with or without recovery following ablative treatment. In this context, it is crucial to identify patients who are most likely to benefit from ablation therapy. For patients with supraventricular arrhythmias, increased cardiac chamber size and the presence of fibrosis are strongly associated with a failure to recover systolic function. These findings suggest that earlier intervention with ablation may improve outcomes in this patient group. For patients with ventricular arrhythmias, key factors influencing the success of ablative treatment include the burden of extrasystolic beats, their origin site, and the presence of ventricular fibrosis. These parameters are critical for identifying patients with the highest likelihood of successful recovery after ablation. While the role of a multiparametric imaging approach—including echocardiography and CMR—has been well established in identifying suitable candidates for ablation, there remain unexplored areas, particularly regarding genetic predisposition. It was hypothesized that differences between responders and non-responders to ablative treatment may be genetically determined. Further research is needed to explore this hypothesis and clarify the genetic factors that influence outcomes.

## Figures and Tables

**Table 1:** Predictors of EF recovery in patients with AIC and supraventricular arrhythmias.

Author, Year	N. Patients	Type of Arrhythmia	Basal EF vs. Follow-Up EF	Duration of Follow-Up	Baseline Predictor of LV Recovery
Luchsinger et al., 1998 [40]	11	AFL	30.9 ± 11.0% vs. 41.4 ± 16.3%	35.5 ± 10.4 months	Higher LVEF and better functional status
Brembilla-Perrot et al., 2016 [45]	103	AFL		13.8 (4.8–33.6) months	No history of IHD, younger age, and no previous use of AAD
Vera et al., 2022 [39]	43	SVT	33.76 ± 9.2% vs. 59.80 ± 5.69%	24 months	QRS < 100 ms, LVEF ≥ 40%, and absence of LGE
Addison et al., 2016 [46]	172	AF	41 ± 6% vs. 49 ± 11%	42 months	Absence of LGE
Aoyama et al., 2024 [23]	129	AF	40.2 ± 7.2% vs. ≥50%	25 (13–40) months	Lower LVEDD and CHA2DS2-VASc
Aoyama et al., 2020 [26]	40	AF/AFL	39.8 ± 8.8% vs. 50.9 ± 10.9%	9.7 ± 8.1 months	Hs-TnT ≤ 12 pg/mL
Aoyama et al., 2023 [51]	90	AF	39.5 ± 6.9% vs. ≥50%	14.2 (10.0–28.0) months	TnI ≤ 11 pg/mL and younger age
Bergonti et al., 2022 [41]	111	AF	32.3 ± 9.4% vs. ≥50%	34 (20–58) months	QRS ≤ 120 ms and lower LAV No persistent AF
Borges-Rosa et al., 2024 [24]	100	AF	36 ± 10% vs. 53 ± 10%	12 months	Narrower QRS and lower LEVDD
Doi et al., 2018 [55]	228	AF	38.1 ± 7.2% vs. 55.7 ± 4.0%	5 months	LowerPTFV1 and Max PWD
Kirstein et al., 2020 [49]	103	AF	33 (25–38)% vs. ≥50%	11 ± 5 months	LA fibrosis ≤ 35–40%
Kusunose et al., 2017 [35]	71	SVT	≤35% vs. ≥50%	6 months	Lower LV size, E/e’, TR-PG, higher LVEF, and RALSR ≤ 0.16
Lee et al., 2021 [36]	60	AFL	43.8 ± 11.8% vs. ≥50%	6 months	LVEDV < 137 mL
Ling et al., 2013 [47]	16	AF	40 ± 10% vs. 60 ± 6%	6 months	Absence of LGE
Marcusohn et al., 2022 [33]	86	AF/AFL	<50% vs. ≥50%	6 months	Higher LVEF, lower LV size, higher HR, and no history of IHD
Moore et al., 2014 [37]	81	SVT	28% (19–39) vs. ≥50%		Lower LV size
Morishita et al., 2023 [25]	72	AF	36 ± 7% vs. 62 ± 7%	12 ± 6 months	LVEDD ≤ 53 mm E’ septal ≤ 6.3 cm/s
Nishikawa et al., 2023 [28]	33	AF	≤50% vs. >50%	12 ± 9 months	Absence of LV-ECV, lower LVEDVLVESV and NT-proBNP
Pizzale et al., 2009 [11]	28	AF	≤40% vs. ≥50%		Higher HR
Prabhu et al., 2017 [48]	68	AF	35.0 ± 9.8% vs. 52.7 ± 11.9%	6 months	Absence of LGE
Stegmann et al., 2022 [38]	134	AF/AFL/AT	39 ± 8% vs. 58 ± 4%	3 months	Lower LVEDV and absence of LGE
Ukita et al, 2021 [44]	81	AF	37.6 ± 8.9%. Δ ≥ 10%	6 months	LVEDD < 53 mm No history of IHD
Yu et al., 2022 [31]	120	AF	41.4 ± 6.4% vs. 58.6 ± 9.9%	19 ± 14 months	Lower LV size HR ≥ 80 bpm LVEF ≥ 40%
Zhao et al., 2024 [27]	80	AF	42 ± 5% vs. 54 ± 9%	6 months	LVEDD < 59 mm Normal voltage zones in LA
Jeong et al., 2008 [34]	21	AF/AFL/SVT	30 ± 11% vs. 58 ± 6%	3 months	Lower LV size
Ichijo et al., 2018 [29]	51	AF	48 ± 12% vs. >50%	13.2 ± 10.9 months	Lower LV size
Kawaji et al., 2021 [30]	280	AF	<40% vs. ≥50% or Δ ≥ 10%	6 months	Lower LVEDD
Yazaki et al., 2020 [32]	140	AF	<50% vs. ≥50% nd/or Δ > 20%	12 months	E’ ≤ 5.4 cm/s LVESV ≤ 98 mL

AAD = anti-arrhythmic drug; AF = atrial fibrillation; AFL = atrial flutter; ECV = extracellular volume; HR = heart rate; Hs-TnT = high sensitivity troponin T; IHD = ischemic heart disease; LA = left atrium; LAV = left atrial volume; LGE = late gadolinium enhancement; LV = left ventricle; LVEDD = left ventricular end diastolic diameter; LVEDV = left ventricular end diastolic volume; LVESV = left ventricular end systolic volume; LVEF = left ventricular ejection fraction; PTFV1 = P-wave terminal force in lead V1; PWD = maximum P-wave duration; RALSR = relative apical longitudinal strain ratio; TnI = troponin I; TR-PG = tricuspid regurgitation pressure gradient.

## Data Availability

Not applicable.
